# Assessment of dental arch stability after orthodontic treatment and oral rehabilitation in complete unilateral cleft lip and palate and non-clefts patients using 3D stereophotogrammetry

**DOI:** 10.1186/s12903-020-01143-1

**Published:** 2020-05-27

**Authors:** Maria Giulia Rezende Pucciarelli, Guilherme Hideki de Lima Toyoshima, Thais Marchini Oliveira, Heitor Marques Honório, Chiarella Sforza, Simone Soares

**Affiliations:** 1 Bauru School of Dentistry/Hospital for Rehabilitation of Craniofacial Anomalies, University of São Paulo, Bauru, São Paulo, Brazil; 2 Department of Pediatric Dentistry, Orthodontics and Public Health, Bauru School of Dentistry and Hospital of Rehabilitation of Craniofacial Anomalies, University of São Paulo, Bauru, São Paulo, Brazil; 3 Department of Pediatric Dentistry, Orthodontics and Public Health, Bauru School of Dentistry, University of São Paulo, Bauru, São Paulo, Brazil; 4 Faculty of Medicine and Surgery, Department of Biomedical Sciences for Health, Functional Anatomy Research Center (FARC), Università degli Studi di Milano, Milan, Italy; 5 Department of Prosthodontics and Periodontology, Bauru School of Dentistry and Hospital of Rehabilitation of Craniofacial Anomalies, University of São Paulo, Alameda Dr. Octávio Pinheiro Brisolla, 9-75, Bauru, São Paulo, 17012-901 Brazil

**Keywords:** Cleft lip, Cleft palate, Dental arch, Three-dimensional imaging, Rehabilitation, Orthodontic appliances

## Abstract

**Background:**

Although arch stability has been studied in patients without a cleft, evidence for patients with a cleft is sparse. Therefore, we compared the dimensions and stability of dental arches in cleft lip and palate patients and those without a cleft.

**Methods:**

Forty participants, 20 with a complete unilateral cleft lip and palate and 20 non-cleft patients aged from 18 to 30 years, with anterior and/or posterior crossbite and receiving orthodontic treatment were evaluated retrospectively. Eighty gypsum casts were digitized using a laser model scanner casts for both groups made immediately after the orthodontic treatment was completed (T1). Also, for the Cleft Lip and Palate group, casts were obtained and digitized 1 year after implant-supported rehabilitation (T2) and for the Non-Cleft Lip and Palate group, 1 year after the conclusion of the orthodontic treatment (T2). The formula: Δ = T2-T1 evaluated the stability of dental arches for inter-canine distances (C-C′), inter-molar distances (M-M’), arch length (I-M), palate surface and volume. The dimensions of the dental arches were measured digitally. The independent *t* test was used for statistical analysis (α = 0.05).

**Results:**

A statistical difference was found in the stability of the groups for inter-canine (cleft area) measurement. At the times T1 and T2, a statistically significant difference was found in the arch length, surface and volume.

**Conclusions:**

This study concluded that in the Cleft Lip and Palate group, the maxillary dimensions were not stabilized after 1 year of orthodontic and prosthodontic treatment (mainly for the inter-canine linear measurement) and that the transverse arch dimensions were smaller compared with those of non-cleft patients.

## Background

Craniofacial anomalies comprise a large group of congenital defects, of which the most prevalent non-syndromic malformation is cleft lip and palate, affecting 1 in 500–700 births, and thus considered a relevant public health problem according to the World Health Organization [1]. Patients with cleft lip and palate (CLP) are identified and typically treated with primary plastic surgeries (cheiloplasty and palatoplasty) usually performed before 12 months of age [2]. Despite rehabilitating esthetics and function, these primary surgeries result in a deleterious effect on maxillary growth [3, 4], with shorter antero-posterior dimension of the alveolar arch in unilateral CLP patients [5]. Rehabilitation is not completed with the anatomic repair of the cleft [2], but requires an interdisciplinary team [2, 6] to achieve anatomic and functional rehabilitation up to skeletal maturity [7, 8].

The treatment of choice to rehabilitate the cleft area is orthodontics [2]. However, the rehabilitative treatment is challenging because the anatomic and functional alterations are directly linked to the malformation type and the age at the beginning the treatment. Obtaining ideal intermaxillary relationships and occlusion, as well as post-treatment stability, requires proper alignment of the teeth in both dental arches, with adequate overjet and overbite [9, 10]. Therefore, many patients require different types of dental prosthesis to rehabilitate the edentulous cleft area, typically the lateral incisor area [11]. Rehabilitating the cleft area with osseointegrated dental implants rather than a fixed dental prosthesis is based on an implant survival rate of 90% at the cleft area [12], similar to that found in patients without a cleft [13].

A systematic clinical documentation protocol comprising all the growth period from birth to adulthood of individuals with oral clefts enables appropriate and prospective planning through tailoring the management required at the many treatment phases [14] and the longitudinal evaluation of the treatment progress [15]. Through study casts, changes in the craniofacial growth can be diagnosed by an analysis of the transversal, anterior-posterior [16, 17] and vertical dental relations [16]. Three-dimensional analysis of dental arches improves data collection [15, 18–22].

Studies comparing the maxillary dimensions after rehabilitative treatment of adult individuals with and without oral clefts are lacking [23]. This information may help to better understand the relevant factors involving the complex rehabilitation of individuals with CLP and to determine parameters for protocols and future research.

This study aimed to compare the post-treatment stability of the linear measurements of adult individuals with a cleft at two phases of the interdisciplinary treatment with those of adults without clefts (18 to 30 years). The null hypothesis was that the post-treatment stability of the maxillary measurements of individuals with oral clefts would be similar to that of individuals without a cleft.

## Methods

This retrospective study followed the Declaration of Helsinki on medical protocol and was approved by the Institutional Review Board regarding ethical aspects under protocol n^o^. 50808215.2.0000.5441 and in accordance with the STROBE guidelines.

A sample size calculation determined that to detect a minimum difference in maxillary width measurement of 0.8 mm, with a standard deviation of 0.7 mm, at a significance level of 5% and a power of 80%, it was necessary to have a minimum of 15 participants per group, based on a pilot study and also from a previous study [24].

Gypsum casts of individuals with and without CLP selected respectively from files of the Hospital for Rehabilitation of Craniofacial Anomalies and Bauru School of Dentistry were digitized using a laser model scanner. The inclusion criteria for both groups were as follows: individuals with or without clefts aged between 18 and 30 years regularly enrolled in each institution with anterior and/or posterior crossbite, who had neither undergone orthognathic surgery nor premolar extraction, who did not wear full denture prosthesis and who had the adequately stored study casts at the study phases. Patients with associated syndromes or malformations or incomplete documentation were excluded.

Forty patients were selected from the 115 individuals evaluated and were divided in two groups: cleft lip and palate (CLP) group (*n* = 20) with complete unilateral cleft lip and palate who had received multidisciplinary treatment at the Hospital for Rehabilitation of Craniofacial Anomalies from birth, with primary surgeries, until the end of treatment when adult, with an implant placed in the cleft area; and the non-cleft group (NCLP), (*n* = 20). A hundred and fifteen patients were assessed in the study, of which 75 (15 CLP and 60 NCLP) did not meet the eligibility criteria because the casts were not suitable for evaluation.

Both groups started and received similar orthodontic treatment lasting approximately 4 years. The long-term analysis was immediately after conclusion of the orthodontic treatment and then 1 year after the complete rehabilitation and orthodontic treatment. The evaluation was done at two times, immediately after removal of the orthodontic appliance and 1 year later, after prosthetic treatment in CLP patients and 1 year later in NCLP patients.

Patients in the CLP group received primary surgery at 3 (cheiloplasty) and 12 (palatoplasty) months of age, respectively. Prior to the secondary alveolar bone graft (mean age 12 years), rapid maxillary expansion was performed, and definitively rehabilitated with prostheses supported by implants in the cleft area after bone growth had ceased. The mean age of the patients was 25.25 ± 3.2 (CLP group).

Rapid maxillary expansion (RME) was performed in both groups with Haas expanders attached to the canines and deciduous second molars and a lingual bar extended to the permanent first molars. The activation protocol was the same for all patients: one full swing per day (2/4 in the morning and 2/4 in the evening) for 7 days. After the active expansion, the device was maintained as retention for 6 months. The present study evaluated the patients immediately after the orthodontic appliance was removed and 1 year later. The stability of the dental arches was the main goal in this groups, if they were maintained or they are not stable, especially in the CLP group rehabilitated with an implant-supported prosthesis.

Eighty gypsum casts were digitized (3Shape R700TM Scanner, Copenhagen, Denmark), and the files were analyzed through the VAM elaboration software (Canfield Scientific, Inc., New Jersey, USA). The evaluation was conducted through 3D images of maxillary arch casts obtained at the following phases for the CLP group - T1: immediately after orthodontic treatment; T2: 1 year after prosthetic rehabilitation and for the NCLP group - T1: immediately after orthodontic treatment; T2: 1 year after orthodontic treatment.

To evaluate the stability of dental arches, the change between T1 and T2 was obtained through the following formula: Δ = T2-T1 for each of the measures: inter-canine distances (C-C′) [18, 25–27], inter-molar distances (M-M’), arch length (I-M) [25, 27, 28], palatal surface and volume [5, 24] (Table 1).

**Table 1:** The measurements of the maxillary dimensions (mm), palate surface (mm^2^) and volume (mm^3^) evaluated

Linear measurements (mm)	Abbreviations	Definition
Inter-canine	C-C′	distance between the cusp tips of the canines.
Intermolar	M-M’	distance between the tips of the mesial-buccal cusps of the first molars.
Arch length	I-M	perpendicular line of the distance between the contact point and the maxillary central incisors up to the intermolar line.
Palate surface	–	Limits standardized: the upper limit was determined by inter-incisor papilla, the lateral limit was surrounded all marginal gingival limits of permanent teeth, and the posterior limit was the midpoint of the distal second molar.
Volume	–	Limits standardized: the upper limit was determined by inter-incisor papilla, the lateral limit was surrounded all marginal gingival limits of permanent teeth, and the posterior limit was the midpoint of the distal second molar.

**Fig. 1:**
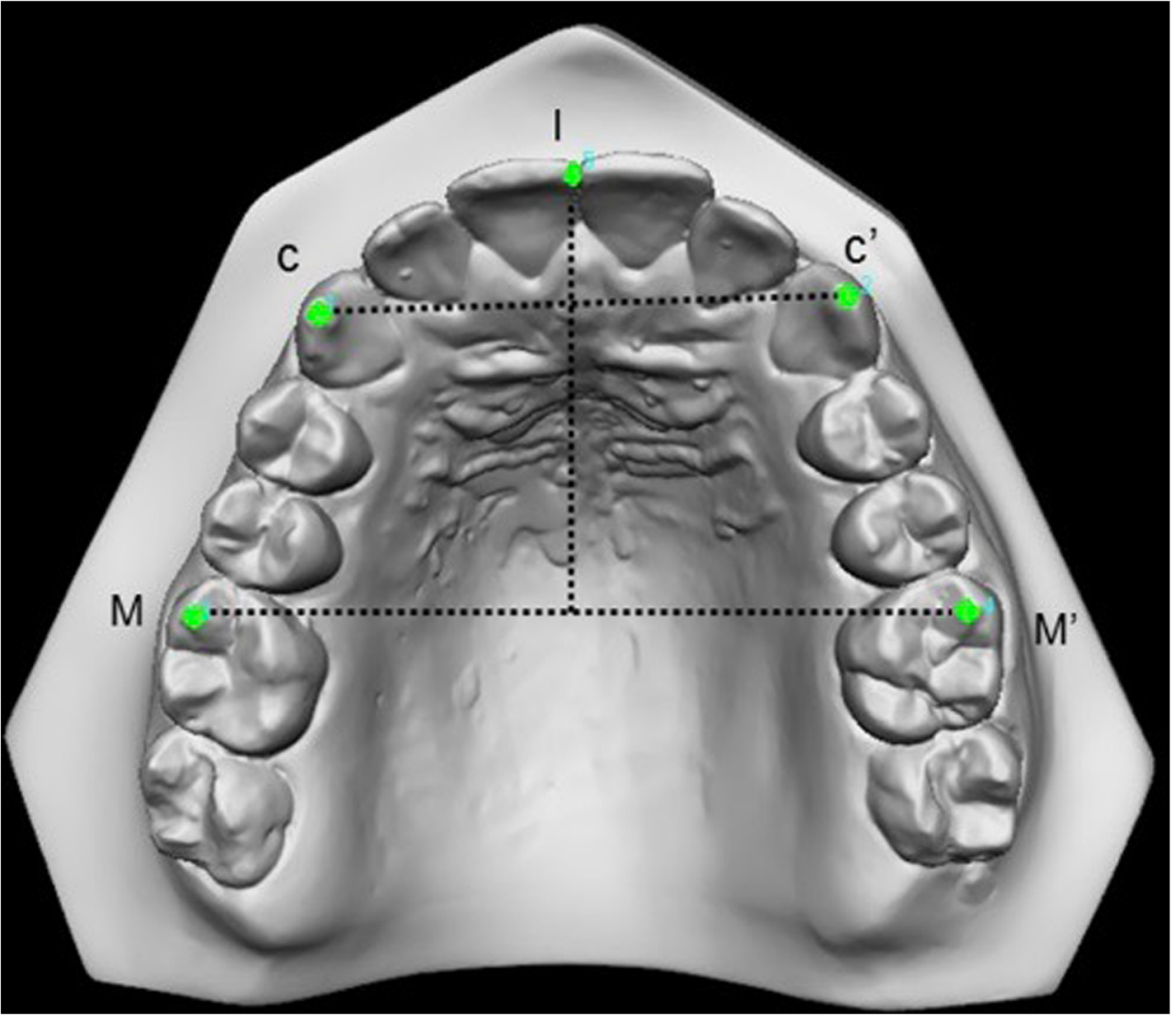
Landmarks points in the maxillary arch for linear measurements (C-C′, M-M’ and I-M)

**Fig. 2:**
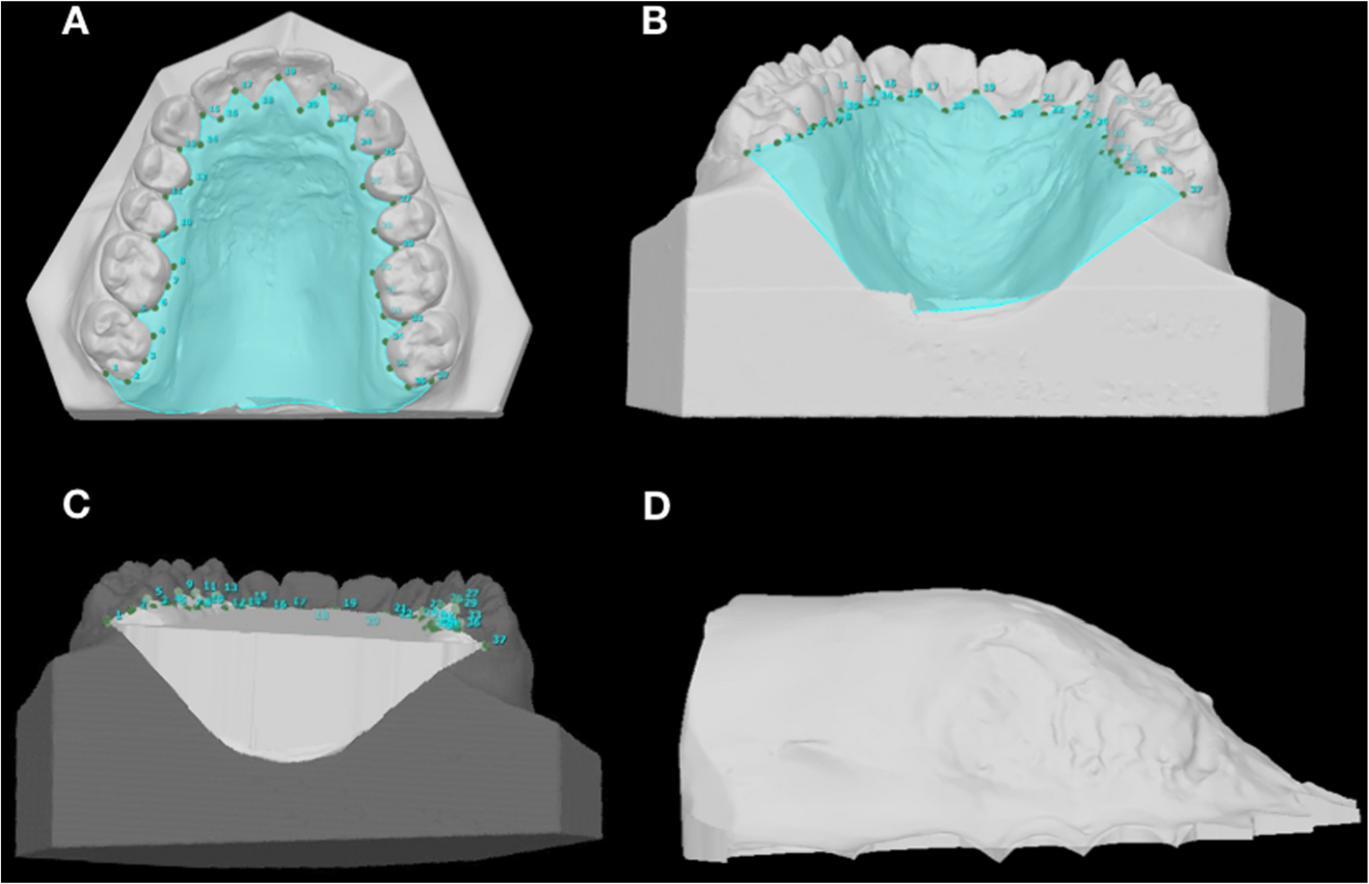
Limits points for palate surface and volume. **a** Delimitate of palatal surface - Limits standardized: the upper limit was determined by inter-incisor papilla, the lateral limit was surrounded all gingival limits of permanent teeth, and the posterior limit was the midpoint of the distal second molar. **b** Delimitate of palate volume. **c** The volume selected. **d** Palate volume

All measurements were made point-to-point by the software that started the acquisition of the image according to Cartesian planes. The anatomic reference points (Fig. 1, Table 1) were used in the maxillary arch to obtain the linear measurements (C-C′, M-M’ and I-M). Limit points for surface and volume were established, although the posterior closure of the palate was done by the system to avoid operator error (Fig. 2).

### Error of method

The measures were evaluated twice by the same examiner in 50% of the digitized models to analyze the error of the method 20 days after the first measurement. The random errors were calculated according to the Dahlberg’ [29] formula (Se^2^ = ∑ d^2^/2n), where Se^2^ is the error variance and *d* is the difference between two determinations of the same variable. The systematic errors were evaluated with the dependent *t* test. No random and systematic errors were found between the measurements obtained on the two different occasions (*p* ≥ 0.05).

### Statistical analysis

The measurements were compared through *t* tests in all cases to evaluate the two groups (CLP and NCLP). The statistical analyses were performed with Sigmaplot version 12.5 (Systat Software Inc., San Jose, CA, USA) (α = 0.05 for all tests).

## Results

The CLP group was composed of 13 women and 7 men; mean age: 25.2 ± 3.2 and the NCLP group of 11 women and 9 men; mean age 22.4 ± 4.6. Immediately after the orthodontic treatment (T1), both groups demonstrated similar measurements, except for the arch length (I-M), palatal surface and volume (Table 2).

**Table 2:** Maxillary dimensions (mm), palate surface (mm^2^) and volume (mm^3^) immediately after orthodontic treatment (T1)

Variables T1	CLP	NCLP		
	Mean ± SD	Mean ± SD	95% CI	*p* (≤ 0,05)
**C-C′**	34.82 ± 5.18	34.62 ± 2.58	−2.32-1.69	0.91
**M-M’**	51.54 ± 3.61	52.08 ± 2.71	2.58–1.50	0.81
**I-M**	26.81 ± 3.81	29.34 ± 2.16	−4.51-0.54	0.01*
**Surface**	19.55 ± 2.80	22.44 ± 3.43	−4.89- 0.87	0.006*
**Volume**	7424.30 ± 2463.02	12,123.90 ± 4043.92	− 6842.96-2556.23	0.001*

*Statistically significant at *p* ≤ 0,05 t-test

*CLP* cleft lip and palate; *NCLP* non-cleft lip and palate; *C-C′,* inter-canine distance; *M-M’* intermolar distance and *I-M* arch length

The individuals with a cleft showed a narrower arch length and smaller three-dimensional measurements (palatal surface and volume) than those without clefts. The same arch shape was maintained after rehabilitation (T2) (Table 3) when compared with Table 2, and the maxillary linear measurements C-C′, M-M’ and I-M decreased in the CLP group.

**Table 3:** Maxillary dimensions (mm), palate surface (mm^2^), volume (mm^3^), 1 year later after rehabilitation/orthodontic treatment (T2)

Variables T2	CLP-R	NCLP-OT		
	Mean ± SD	Mean ± SD	95% CI	*p* (≤ 0,05)
**C-C′**	32.28 ± 5.52	34.60 ± 2.65	−2.87-0.98	0.40
**M-M’**	50.62 ± 3.24	51.71 ± 2.92	−3.06-0.88	0.13
**I-M**	26.26 ± 3.14	29.02 ± 2.37	−4.53-0.97	0.003*
**Surface**	18.63 ± 3.55	22.16 ± 2.62	−5.53-1.53	0.001*
**Volume**	7297.50 ± 5351.00	11,725.0 ± 4802.50	− 6233.64-2424.05	0.001*

*Statistically significant at *p* ≤ 0,05; t-test

*CLP* cleft lip and palate; *NCLP* non-cleft lip and palate; *C-C′,* inter-canine distance; *M-M’* intermolar distance and *I-M* arch length; R, rehabilitation; OT, orthodontic treatment

The evaluation of stability (Δ) between the study phases (T2-T1) showed statistically significant differences between groups for the inter-canine measurement (C-C′) (Table 4). The CLP group exhibited a proportional reduction in the transversal measurement, but the intermolar (M-M’) and arch length measurement (IM) showed no statistically significant difference between the groups. A proportional variation in the linear measurements without statistically significant differences was observed for the NCLP group. Negative (−) values indicated a reduction in the maxillary dimensions.

**Table 4:** Stability (Δ = T2-T1) of the maxillary dimensions (mm), palate surface (mm^2^) and volume (mm^3^) between groups

Variables	CLP	NCLP		
	Mean ± SD	Mean ± SD	95% CI	P (≤ 0,05)
**ΔC**	−0.42 ± 1.25	0.10 ± 0.02	−1.55-0.30	0.01*
**ΔM**	−0.47 ± 1.01	−0.40 ± 1.02	−1.37-0.27	0.35
**ΔIM**	−0.35 ± 1.19	−0.25- ± 0.75	−1.07-0.63	0.69
**Surface**	−0.92 ± 1.89	−0.27 ± 2.52	−2.07-0.78	0.36
**Volume**	420.85 ± 1723.18	50.10 ± 2151.25	− 876.94-1618.44	0.55

*Statistically significant at *p* ≤ 0,05 t-test

*CLP* cleft lip and palate; *NCLP* non-cleft lip and palate; *ΔC* inter-canine distance; *ΔM* intermolar distance and *ΔIM*, arch length

## Discussion

The study compared maxillary dimensional alterations in adult individuals with CLP from the Hospital for Rehabilitation of Craniofacial Anomalies at two treatment phases (after orthodontic treatment and after implant-supported prosthesis delivery) with those without CLP but receiving orthodontic treatment. The methodology, which was based on the relevant literature and used a digitized cast and a 3D analysis, resulted in a significant change in the data collected [15, 18–22].

Individuals with oral clefts underwent a long interdisciplinary treatment [2, 6, 8, 30, 31] until skeletal maturity [7, 8] to rehabilitate the anatomic and functional deficiencies. The treatment protocol in the Hospital for Rehabilitation of Craniofacial Anomalies consisted of corrective primary surgeries during childhood, usually performed before 12 months of age [2], pediatric follow-up until adolescence, orthodontic evaluation and determination of the growth pattern, usually due to surgical interventions at childhood. These individuals with oral clefts had a Class III skeletal pattern caused by the maxillary sagittal deficiency, and maxillary expansion treatment is often necessary before secondary alveolar bone grafting [2, 24]. Ayub et al. (2016) compared the dentoalveolar effects of RME in individuals with and without clefts and found similar maxillary expansion results for both groups [24]. Thus, the comparison between adult individuals with and without CLP after orthodontic treatment (T1) was justified.

Individuals with CLP commonly have agenesis of the maxillary lateral incisor. The orthodontic treatment in individuals with CLP is complex and many factors should be carefully evaluated. Nevertheless, orthodontic treatment is the first choice for rehabilitating the edentulous space of the lateral incisor [2]. However, other options for the rehabilitation of the cleft area may be necessary, including different types of prosthesis [11, 32].

Studies on adult individuals with CLP and long-term studies on the stability of rehabilitation/prosthetic treatment comparing individuals with and without oral clefts [23] are lacking. The digitized casts obtained during the treatment phases can provide more information on the treatment stability of individuals with oral clefts.

3D digital images have advantages, including rotation and manipulation similar to those of gypsum casts [31, 33, 34], accurate measurements, improved handling and storage, planning and execution of the various stages of rehabilitation treatment [21, 22]. These advantages were important in this study, since two treatment phases were considered (T1 and T2). Moreover, the study of these phases enabled the long-term evaluation of the stability of the maxillary dimensions during treatment.

Landmarks have been used to perform the linear measurements for evaluating the maxillary dimensions and changes over time in individuals with a cleft lip and palate from an early age to assess the growth and maxillary bone development [18, 25, 26, 28, 33–36]. In this present study, we used strategic landmarks for the evaluation of maxillary dimensions at the permanent dentition: inter-canine distance (C-C′) [3], intermolar distance (M-M’) [3], arch length (I-M) [24], palatal surface and volume [5] (Table 1).

The cleft typically affects the alveolar ridge, causing tooth agenesis (generally the lateral incisor) [2] and requiring prosthetic treatment. However, this study only evaluated patients who received implants in the cleft area, enabling a comparison between groups. Both groups were selected to enable the analysis of the inter-canine, intermolar and arch length measurements. This would not have been possible if the sample had included individuals with clefts in whom the canine was mesialized to close the lateral incisor space. Because of this, our sample was a convenience sample. When a treatment is concluded in a CLP patient, regardless of dental treatment, the parameters or gold standard are based on NCLP patients, and therefore these two groups were compared.

The intergroup stability comparison revealed a statistically significant difference in the inter-canine measurement (C-C′), and the dimensional alteration demonstrated a reduction of − 0.42 mm during the study period. Notably, the non-stabilized area is near the cleft after 1 year of rehabilitation (Table 4), while the other measurements analyzed demonstrated stability. As expected, the cleft area is the most vulnerable to orthodontic relapse.

At T1 (immediately after the orthodontic treatment), individuals with CLP had a lower arch length (I-M) than non-cleft individuals, while inter-canine (C-C′) and intermolar measurements (M-M’) were not statistically different between groups (Table 2). This result is consistent with that of Ayub et al. [24] and Athanasiou et al. [35], who reported that the arch length of individuals with CLP is smaller than that of individuals without CLP from the deciduous to the permanent dentition [24, 35]. The analysis of the linear measurements’ stability (T2-T1) revealed that non-cleft individuals exhibited a proportional stability in all measurements, without statistically significant differences (Table 4). In contrast, individuals with CLP showed statistically significant difference in the inter-canine (C-C′) measurement (Table 4), with a reduction of − 0.42 mm. Similarly, Li and Lin (2007) assessed the post-treatment stability of individuals with a cleft and concluded that relapse occurred to some extent after orthodontic treatment and arch width decreased after retention [3].

The present study also found a statistically significant difference in the palatal surface (T1 *p* = 0.006/ T2 *p* = 0.001) and volume (T1/T2 *p* = 0.001) between the groups at both times studied. Rusková et al. (2014) reported similar results when comparing palatal volume in unilateral cleft and non-cleft patients and concluded the average of volume in the CLP group was shallower, narrower, shorter and more asymmetrical [5]. These findings were supported by other studies which determined that palate width as a whole was reduced compared with controls [36, 37] CLP individuals typically have smaller palate surfaces than non-cleft individuals [38], which suggests that there is an intrinsic tissue deficiency in the palate/maxilla before palatoplasty (Lo et al., 2003) and that after the primary surgeries, the dental arch narrows [39].

These dimensional alterations in the maxillary arch of individuals with a cleft are a challenge for the rehabilitation treatment, which requires periodic follow-ups after orthodontic treatment and implant installation to check the prosthesis and the occlusion. Occlusal adjustments are often necessary in individuals with CLP, a fact that can be explained by the dental changes occurring over time, as identified by this study. It is important to emphasize that the relapse of the upper arch in CLP patients it is not enough to cause posterior crossbite after 1 year, and the alterations can be seen in an anterior arch with regular occlusal adjustments. Whether the implant-supported prothesis influences the arch stabilization is unclear. The results of the present study suggest that rehabilitation with implants did not stabilize the dimensions of the maxillary arches, but more studies are necessary with different types of prothesis to determine the outcome.

Limitations of the study included not evaluating orthodontic relapse, since only the maxillary dental arches were assessed. An evaluation of relapse should include bone development and occlusion, which was outside the scope of this investigation. An additional limitation was the convenience sample, because the eligibility criteria were so specific.

This study was conducted on individuals treated at one rehabilitation center, and thus, the results should be extrapolated with caution. Intercenter studies are necessary to illuminate the growth and stability of the rehabilitative treatment in individuals with a cleft lip and palate.

## Conclusion

In the CLP group, the maxillary dimensions were not stabilized after 1 year with orthodontic and implant-supported prostheses as compared with non-cleft patients, and the palate surface and volume were smaller compared with non-cleft patients. Oral rehabilitation specialists should be careful when treating this kind of patient.

## Data Availability

The datasets used and/or analyzed during the current study are not publicly available but are available from the corresponding author on reasonable request.

## Abbreviations

CLP: Cleft lip and palate
NCLP: Non-cleft lip and palate
RME: Rapid maxillary expansion
T1: Immediately after orthodontic treatment
T2: 1 year after prosthetic treatment
C-C′: Inter-canine distance
M-M’: Inter-molar distance
I-M: Arch length
